# Discovery of *PHB1* as a Novel Candidate Gene in Dominant Optic Atrophy

**DOI:** 10.1111/cge.70174

**Published:** 2026-05-01

**Authors:** Marija Volk, Aleš Maver, Martina Jarc Vidmar, Nuša Trošt, Tanja Višnjar, Ana Fakin, Lea Kovač, Maja Šuštar Habjan, Lucija Malinar, Sanja Petrović Pajić, Urška Dragin Jerman, Rok Romih, Marko Hawlina, Borut Peterlin

**Affiliations:** ^1^ Clinical Institute of Genomic Medicine, University Medical Centre Ljubljana Ljubljana Slovenia; ^2^ Faculty of Medicine University of Ljubljana Ljubljana Slovenia; ^3^ Eye Hospital, University Medical Centre Ljubljana Ljubljana Slovenia; ^4^ Clinic for Eye Diseases, University Clinical Centre of Serbia Belgrade Serbia; ^5^ Faculty of Medicine University of Belgrade Belgrade Serbia; ^6^ Faculty of Medicine Institute of Cell Biology, University of Ljubljana Ljubljana Slovenia

## Abstract

Hereditary optic neuropathies comprise a genetically heterogeneous group of disorders caused by pathogenic variants in mitochondrial and nuclear genes. Despite increasing diagnostic yields, many patients remain without a molecular diagnosis. We report a novel candidate heterozygous variant in the *PHB1* (Prohibitin 1) gene in a large family affected by autosomal dominant optic atrophy. A three‐generation family with slowly progressive visual acuity loss due to optic neuropathy and an apparent autosomal dominant pattern was clinically characterized and recruited for genetic counseling. Exome sequencing and genome‐based linkage mapping were performed, alongside protein modeling and in vitro experiments to obtain functional evidence. Family‐based whole‐genome linkage mapping identified a heterozygous missense variant, c.440C>T (p.Ser147Phe), in *PHB1* in all five affected individuals. The variant substitutes p.Ser147Phe within an evolutionarily conserved alpha‐helix domain of PHB1, a mitochondrial protein with multiple roles. In silico modeling suggested that p.Ser147Phe may disrupt PHB1 stability and function through loss of hydrogen bonding, steric hindrance, and altered hydrophobic interactions. In vitro experiments suggested potential alterations in mitochondrial dynamics in variant carriers, including a changed ratio of L‐OPA1 to S‐OPA1 compared with non‐carriers. We present initial evidence that *PHB1* is a novel candidate gene potentially associated with dominant optic atrophy or a related mitochondrial disorder. This represents the first report implicating *PHB1* in a Mendelian disease. Further studies are required to validate this association.

## Graphical abstract

A heterozygous PHB1 missense variant (p.Ser147Phe) segregates with autosomal dominant optic atrophy in a multi‐generation family. Structural and cellular analyses suggest altered mitochondrial dynamics, identifying PHB1 as a novel candidate gene for hereditary optic neuropathy.

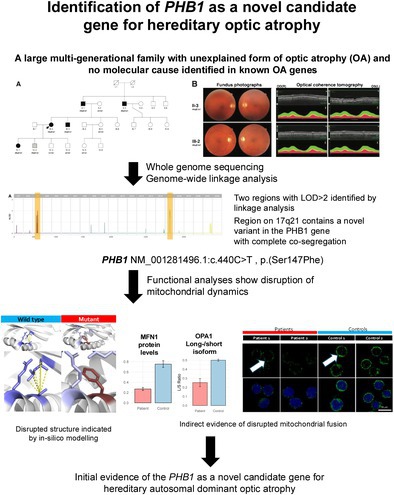

## 1 Introduction

Hereditary optic neuropathies are a genetically heterogeneous group of disorders caused by pathogenic variants in one of 27 recognized nuclear or mitochondrial genes [1]. Experience shows that a specific genetic diagnosis related to one of these genes can be established in 20%–30% of cases, while the majority remain unexplained [2]. All genes discovered to date that are involved in the pathogenesis of optic atrophy encode mitochondrial proteins [2].

A three‐generation family, in which four members were diagnosed with optic neuropathy in early adulthood, was referred for genetic counseling and testing. The pedigree indicated an autosomal dominant inheritance. Whole‐exome and mitochondrial sequencing revealed no pathogenic variants in any of the known genes. Whole‐genome sequencing with linkage mapping identified an extremely rare missense variant in the *PHB1* gene. As the role of *PHB1* in the context of Mendelian disease is not yet clear, we performed in vitro functional studies to support its causality. In this study, we present initial evidence that the potential cause of dominant optic atrophy in this family is a missense variant in the *PHB1* gene encoding a mitochondrial protein, prohibitin 1.

## 2 Methods

The study was performed on a Slovenian three‐generation family with four affected members with optic neuropathy. Extensive ophthalmological evaluations, including clinical examinations with visual acuity and color vision assessment, visual fields, fundus photography, optical coherence tomography, and electrophysiology testing, were performed at UMC Ljubljana Eye Hospital from 2006 to 2025. Written informed consent was obtained from all participants in accordance with the principles of the Declaration of Helsinki.

Exome sequencing (ES) was carried out in four available affected members, whereas genome sequencing (GS) with linkage mapping was conducted for four available affected and five unaffected family members. Functional evaluation of the identified variant was conducted via in silico homology modeling and in vitro experiments.

## 3 Results

**FIGURE 1:**
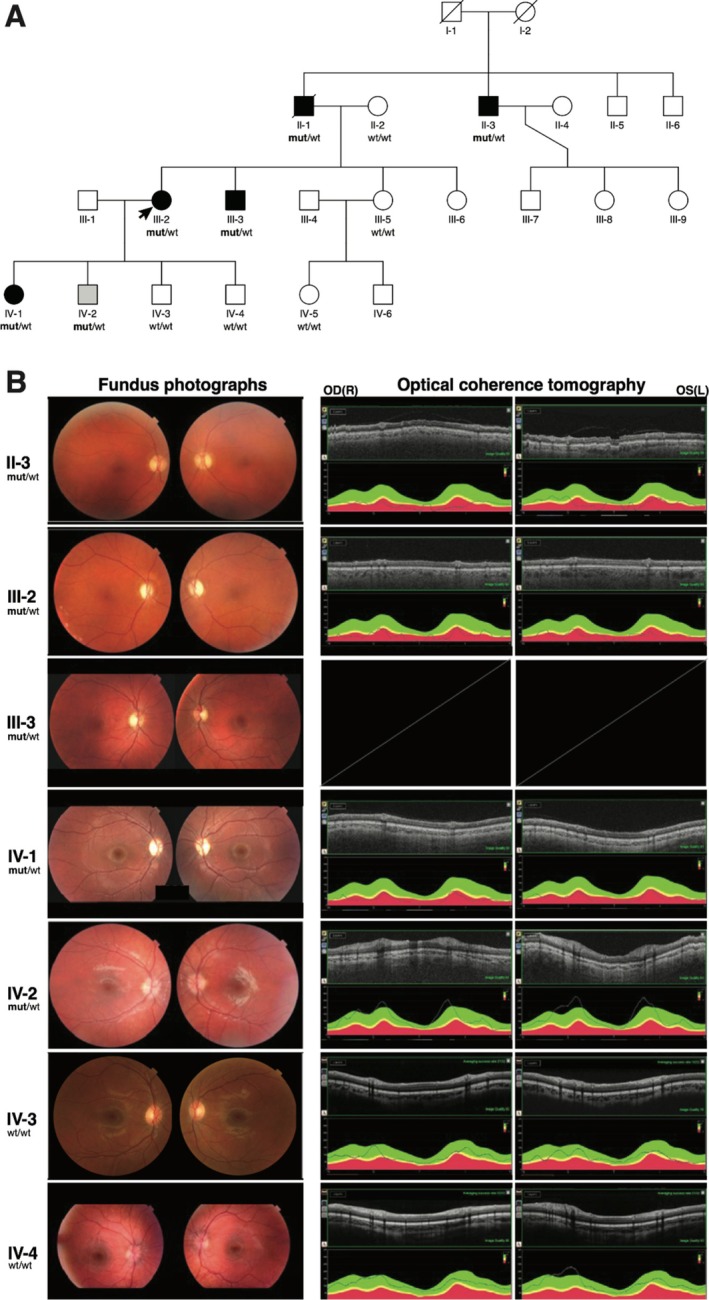
Family pedigree with multiple members affected by optic atrophy, along with retinal imaging of family members who underwent an ophthalmic evaluation. (A) Symbols in black represent affected individuals, while clear symbols indicate unaffected individuals. Fundus pictures and OCT images were taken from their last ophthalmology exam (the age of the patient upon imaging can be seen in Table 1), except for patient III‐3 where fundus image without OCT from the beginning of follow‐up is shown. A gray symbol is used for an individual (IV‐2) who was examined in 2025 at the age of 5 and did not yet show definitive signs of optic atrophy, although the discs were slightly paler than would be expected at this age. On his OCT image, retinal ganglion cells were thinned in the macular area, which is an early indication of impending optic neuropathy (Figure S6). The squares stand for males and the circles for females. The crossed‐out symbols represent deceased individuals. Tested members are marked PHB1‐positive if carrying the PHB1 variant and PHB1‐negative if not. A detailed clinical description for deceased member II‐1 was not available. The abbreviations P1, P2, and C1 used in text refer to family members IV‐1, III‐2 and IV‐4, respectively. (B) Fundus photography and OCT RNFL scans of both eyes (OD‐right and OS‐left) are shown for available family members.

The pedigree of the studied family and the fundus photography with OCT scans of the retinal nerve fibre layer (RNFL) are displayed in Figure 1. Detailed clinical characteristics of the individuals included in the study are summarized in Table 1 and further elaborated in Supporting Information. All affected patients showed slowly progressive bilateral visual acuity loss, with impaired color vision, central scotoma in the visual fields, slowly progressive retinal nerve fiber layer thinning on OCT, and signs of optic neuropathy on electrophysiology testing: abnormal N95 wave of pattern ERG (PERG) and abnormal visual evoked potentials (VEP).

**TABLE 1:** Clinical ophthalmic and other clinical characteristics of the family members.

Family member	Registry ID	Genetic findings	YOB	Sex	Age at onset	Age at exam	VA RE Snellen	VA LE Snellen	Ishihara RE	Ishihara LE	Visual field RE, LE	Optic disc	OCT RNFL	OCT macula	PERG	Pattern‐reversal VEP	Additional findings
II‐3	0014	mut/wt	1958	M	40	61	0.1 cc	CF 1.5 m	0/15	0/15	Central scotoma	Diffuse pallor	RNFL atrophy	RGC atrophy	Slightly abnormal P50 (loss of fixation), abnormal N95	Abnormal (reduced, delayed)	
						63	CF 1.5 m	CF 1 m	0/15	0/15	Central scotoma	Diffuse pallor	RNFL atrophy	RGC atrophy			
II‐2		wt/wt		F	No problems												
III‐2 (P2)	0066	mut/wt	1976	F	8	34	0.2 cc	0.2 cc	2/15	2/15		Diffuse pallor	RNFL atrophy		Abnormal N95	Abnormal (reduced, delayed, bifid wave‐shape)	
						44	0.1 cc	0.1 cc	1/15	1/15	Central scotoma	Diffuse pallor	RNFL atrophy	RGC atrophy			
III‐3	0015	mut/wt	1981	M	10	33	0.4	0.4	1/15	1/15		Temporal pallor	RNFL atrophy		Abnormal N95	Abnormal (delayed)	
						35	0.32	0.2									Sensorineural hearing impairment
IV‐1 (P1)	1089	mut/wt	1998	F	8	8	0.8	0.8				Temporal pallor			Normal	Abnormal (delayed)	
						16	0.6	0.4	11/15	9/15	Central scotoma	Temporal pallor	RNFL atrophy		Abnormal N95	Abnormal (delayed, partially reduced)	
						23	0.4	0.3			Central scotoma	Diffuse pallor	RNFL atrophy	RGC atrophy			
IV‐2	1167	mut/wt	2019	M		5	1.0	1.0	15/15	15/15		Normal	RNFL normal	Paracentral RGC thinning			
IV‐3	1090	wt/wt	2001	M	No problems	15	1.0	1.0	15/15	15/15	Normal		Borderline RNFL		Normal	Normal	
IV‐4 (C1)	1091	wt/wt	2007	M	No problems	9	1.0	1.0	13/15	15/15	Normal		RNFL normal				

*Note*: Based on affected individuals, the earliest sign of the disease was thinning of RGC layer in macular OCT followed by RNFL layer thinning and progressive disc pallor from the temporal side. Visual acuity was normal at the age of 5, slightly subnormal (0.8) at the age of 8, and gradually decreasing during the lifespan of other affected members, reaching approximately 0.2– 0.4 in the 30s, falling to 0.1 in the 40s, and reaching CF in the 60s. Progressive central scotoma and color vision loss, and electrophysiological signs of optic neuropathy (progressive decline of PERG N95 wave and VEP P100 wave) were seen. Except for one patient who had sensorineural hearing impairment, there were no other multisystemic features.

Abbreviations: CF, counting fingers; F, female; LE, left eye; M, male; OCT, optical coherence tomography; PERG, pattern electroretinography; RE, right eye; RGC, retinal ganglion cells; RNFL, retinal nerve fiber layer; VA, visual acuity; VEP, visual evoked potentials; YOB, year of birth.

**FIGURE 2:**
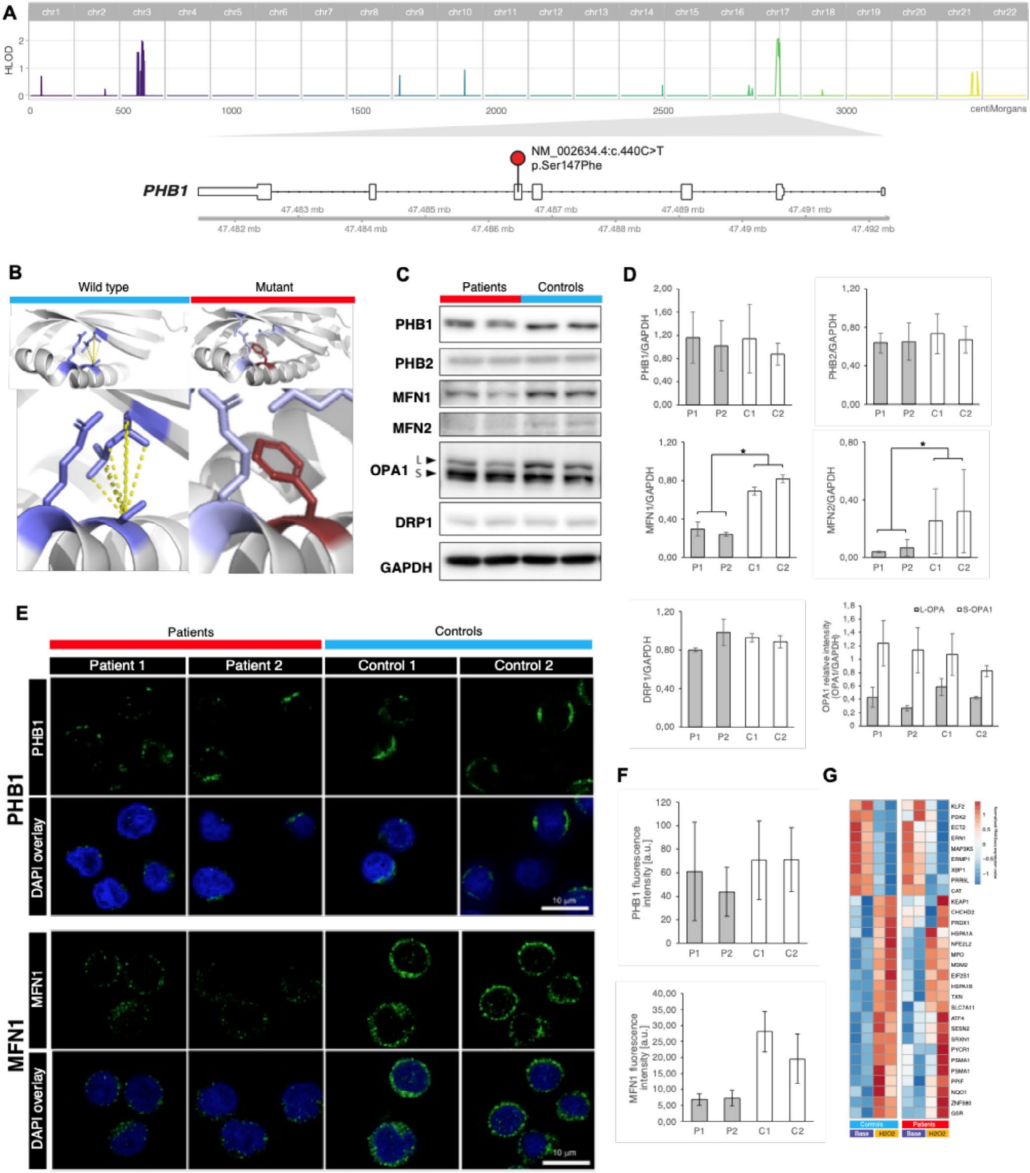
In vitro functional characterization of missense variant p.Ser147Phe in the *PHB1* gene. (A) The results of the genome‐sequencing‐based linkage scan in the family. Two regions with LOD scores over 2 were identified, with the region on chromosome 17 containing the *PHB1* gene and an ultrarare missense variant *PHB1* (NM_002634.4):c.440C>T (p.Ser147Phe). (B) Structural modeling of the PHB1 protein: On the left—wild type structure and on the right a PHB1 structure with Serine at position 147 replaced by Phenylalanine (red). Amino acid substitution occurs within a critical alpha‐helix of the PHB1 protein, important for lipid raft associations and protein–protein interactions (PMID: 17427253). Based on the molecular modeling, yellow dashed lines indicate possible hydrogen bond interactions in the wild‐type structure (left model). (C) Western blot analysis of PHB1, PHB2, MFN1, MFN2, OPA1, and DRP1 protein levels in PBMCs obtained from 2 subjects with PHB1 variant (P1, P2) and 2 subjects without the variant (C1, C2‐unrelated individual), where GAPDH was used as a loading control. Representative immunoblots are shown. (D) Immunoblots were quantified by ImageJ, and data are presented as mean ± SD of at least 2 replicates. Significance was tested using a two‐tailed Student's test. *p < 0.05. (E) Representative immunofluorescence images of PHB1 and MFN1 in PBMCs from individuals with (P1, P2) and without the PHB1 variant (C1, C2). The cell nuclei are stained with DAPI. (F) Quantification of mean ± SD fluorescence intensity of PHB1 and MFN1 by ImageJ software. The third plot in this panel displays intracellular PHB1 fluorescence heterogeneity in PBMCs quantified as the coefficient of variation (CV) of PHB1 pixel intensities within individual cells. (G) Results of RNAseq‐based analysis of the transcriptional response to oxidative stress. On the left, 30 genes with the most pronounced transcriptional response in wild‐type cells in the oxidative stress pathway are presented. On the right, the transcriptional response of these genes is shown in cells harboring the p.Ser147Phe variant, suggesting a possible difference in the normal transcriptional response to oxidative stress.

ES analysis in affected family members (II‐3, III‐2, III‐3, and IV‐1) revealed no causal variants in genes associated with optic atrophy. No pathogenic mitochondrial genome variants, even at lower levels of heteroplasmy, were detected—despite the coverage of mitochondrial genome exceeding 2.000× in all patients analysed with whole‐genome sequencing. Whole‐genome linkage mapping identified a heterozygous missense variant NM_002634.4:c.440C>T (p.Ser147Phe) in the *PHB1* gene that co‐segregated with optic neuropathy in affected members (Figure 1, Figure S1). This variant, which has not been previously reported, results in an amino acid substitution from serine to phenylalanine at position 147 in the evolutionarily conserved domain of the PHB1 protein (Figure 2A). In silico prediction tools indicated this variant as pathogenic (Table S1). Homology modeling also indicated that the bulkier phenylalanine side chain might reduce hydrogen bonding and destabilize the PHB1 protein structure (Figure 2B).

To support its potential functional relevance, we conducted functional in vitro analyses under normal and oxidative stress conditions. Western blot (WB) analysis showed that PBMCs from patients (P1, P2) exhibited a broader, less intense PHB1 band compared to the discrete, more intense signal observed in controls (C1, C2) (Figure 2C). This pattern persisted following H_2_O_2_ exposure (Figure S2). However, no significant differences in quantity were observed between the groups (Figure 2D). Confocal microscopy showed no significant differences in fluorescence intensity of PHB1 between patient and control samples, although the protein distribution differed. In controls, the PHB1 signal appeared more spatially confined, with a tendency toward perinuclear enrichment, whereas in patient cells PHB1 showed a more heterogeneous cytoplasmic distribution, consistent with the increased coefficient of variation observed in patients (Figure 2F). Furthermore, WB analysis revealed that the levels of the fusion proteins mitofusin 1 and 2 (MFN1 and MFN2) were significantly reduced in the PBMCs of patients compared to controls (Figure 2C,D). A similar reduction in MFN1 intensity was confirmed by immunofluorescence staining (Figure 2E,F).

We also observed enhanced proteolytic processing of OPA1 in PBMCs from patients compared to controls, with the ratio of long to short OPA1 isoforms (L‐/S‐OPA1) shifted toward the S‐OPA1 isoform (Figure 2C,D). The total OPA1 protein level did not differ significantly, and no differences were observed in the levels of total DRP1 and PHB2 proteins between patient and control cells (Figure 2C,D). Notably, the fluorescence intensity of the DRP1 signal was significantly lower in both patient samples compared to the controls (Figure S3). Furthermore, the transcriptome analyses results suggested that the response to oxidative stress may be altered in cells harboring the mutation, although no genes surpassed the significance threshold after applying the correction for multiple testing (Figure 2G).

Additionally, transmission electron microscopy and mtDNA quantification (Figures S4 and S5) revealed no significant differences between patient and control samples (Supporting Information).

## 4 Discussion

We present initial evidence of *PHB1* as a novel candidate gene associated with dominant optic neuropathy with slowly progressive visual acuity loss and all signs of optic neuropathy in a large three‐generation family. GS mapping identified a variant c.440C>T in the *PHB1* gene, which segregates with dominant optic atrophy in affected family members. GS‐based mapping identified a variant c.440C>T in the *PHB1* gene, which segregates with dominant optic atrophy in all affected family members. To our knowledge, *PHB1* has not been previously linked to optic neuropathy or any other Mendelian disease. In the mitochondrial inner membrane, PHB1 partners with PHB2 to form hetero‐oligomeric complexes and plays a role in mitochondrial biogenesis, stability, and dynamics in collaboration with OPA1, MFN1, MFN2, DRP1, and other proteins [3, 4, 5].

In silico modeling suggests that p.Ser147Phe substitution in the *PHB1* gene may disrupt hydrogen bonding, potentially causing structural instability. Our WB results show an altered PHB1 pattern, suggesting altered post‐translational processing. Additionally, we observed reduced levels of MFN1 and MFN2 and a shift in the L‐/S‐OPA1 ratio toward the short isoform in patient compared to control cells. These findings are compatible with the altered mitochondrial fusion dynamics, although they represent indirect markers of mitochondrial function rather than direct evidence of dysfunction. While mitochondrial morphology remained unchanged, our results suggest that the p.Ser147Phe PHB1‐variant may affect mitochondrial dynamics, possibly through an imbalance between mitochondrial fusion and fission. This process has already been implicated in the pathogenesis of several hereditary optic neuropathies [6, 7, 8]. However, the exact mechanism by which this variant contributes to the disease remains to be fully elucidated. While we recognize that the moderate linkage signal and suggestive functional data are not sufficient to confirm the role of PHB1 in optic atrophy, the evidence collected in this study provides compelling initial evidence to support its role as the candidate gene until further patients are reported and more extensive functional analyses are performed.

In conclusion, the study results provide initial evidence that *PHB1* is a novel candidate gene for AD optic atrophy, but the precise mechanism by which the c.440C>T (p.Ser147Phe) variant in the *PHB1* gene affects the protein and its function remains to be elucidated. According to the observation in the present family, the clinical course is similar to that of *OPA1*‐positive DOA [1].

## Author Contributions

M.V., N.T., T.V., L.M., M.J.V. and A.M. drafted the manuscript. N.T. and T.V. performed functional studies. U.D.J. and R.R. performed microscopic and cellular analyses. L.M. performed in silico modeling. M.J.V., A.F., L.K., M.Š.H., S.P.P., and M.H. performed ophthalmological and neuro‐ophthalmological assessments. All authors contributed to data interpretation and reviewed and critically revised the manuscript. M.H. and B.P. supervised the study. All authors approved the final version of the manuscript.

## Funding

The study was in part supported by the Slovenian Research Agency (ARIS programmes P3‐0108, P3‐0333 and P3‐0326). [Correction added on 20 May 2026, after first online publication: ‘P3‐0108’ has been added.]

## Disclosure

The first author of the study, Dr. Marija Volk, sadly passed away during the preparation of the manuscript. We dedicate this study to a lasting memory of her dedication to genetics and her tireless work in the field of genetic ophthalmologic diseases.

## Conflicts of Interest

The authors declare no conflicts of interest.

## Supporting information


**Figure S1:** Genome‐wide linkage analysis scan (A) Results of the genome‐sequencing‐based linkage scan in the family. An interval on chromosome 17 (chr17: 36.5–47.5 Mb) with a maximum LOD score of 2.1 was identified by analysis of co‐segregating SNP markers. (B) The region on chromosome 17 with the highest linkage score was surveyed for candidate genes and variants, including the PHB1 gene. (C) A heterozygous missense variant c.440C>T (p.Ser147Phe) in exon 4 was observed to perfectly cosegregate with the optic atrophy in all surveyed family members.
**Figure S2:** Immunoblots of PHB1, PHB2, MFN1, MFN2, OPA1, DRP1 and GAPDH in PBMCs. Representative immunoblots of PHB1, PHB2, MFN1, MFN2, OPA1, DRP1, and GAPDH in PBMCs from 2 individuals with optic atrophy, who carry PHB1 variant and 2 controls before (0 h) and after H_2_O_2_ exposure for the indicated time periods (6 h, 24 h).
**Figure S3:** Immunofluorescence images of PHB2, DRP1 and TOMM20 in PBMCs. The figure displays representative immunofluorescence images of PHB2, DRP1, and TOMM20 in PBMCs from individuals with (P1, P2) and without the PHB1 variant (C1, C2). The fluorescence intensity and the signal distribution of PHB2 show no significant differences between individuals with (P1, P2) and those without the PHB1 variant (C1, C2). The same observation applies to TOMM20 immunofluorescence in the same samples. The fluorescence intensity of DRP1 is lower in both individuals with the PHB1 variant (P1, P2) compared to the control individuals without the PHB1 variant (C1, C2), while the distribution pattern remains comparable across samples. In the same samples, we also observe a lower fluorescence intensity of the TOMM20 signal in individuals with the PHB1 variant (P1, P2) compared to the control individuals without the PHB1 variant (C1, C2). There are no differences in the distribution of the TOMM20 signal between the samples.
**Figure S4:** TEM imaging of mitochondria. TEM imaging was performed in patients who carry the PHB1 variant (P1 and P2) and controls without the variant (C1 and C2). The ultrastructure of the mitochondria shows no significant differences between the patients and controls.
**Figure S5:** Relative mitochondrial DNA copy number estimation. Relative mitochondrial DNA copy number was estimated using RT‐qPCR measurement of mitochondrial D‐loop and ND1 (relative to nuclear B2M gene) in PBMCs from 2 subjects carrying the PHB1 variant (P1, P2) and two control subjects without the variant (C1, C2), both before and after inducing oxidative stress with 1000 μM H_2_O_2_ for 6 h.
**Figure S6:** OCT showing thinned retinal ganglion cells in the macular area in the patient IV‐2 at the age of 5 years.
**Table S1:** In silico prediction tools.

## Data Availability

The data that support the findings of this study are available on request from the corresponding author. The data are not publicly available due to privacy or ethical restrictions.
