# Supplementary material for: Discovery of PHB1 as a Novel Candidate Gene in Dominant Optic Atrophy
Source: Clin Genet. 2026 May 1;110(2):165–71. doi: 10.1111/cge.70174 (PMC13327145; doi:10.1111/cge.70174)
Supplement: Supplementary file 1 — Figure S1: Genome‐wide linkage analysis scan (A) Results of the genome‐sequencing‐based linkage scan in the family. An interval on chromosome 17 (chr17: 36.5–47.5 Mb) with a maximum LOD score of 2.1 was identified by analysis of co‐segregating SNP markers. (B) The region on chromosome 17 with the highest linkage score was surveyed for candidate genes and variants, including the PHB1 gene. (C) A heterozygous missense variant c.440C>T (p.Ser147Phe) in exon 4 was observed to perfectly cosegregate with the optic atrophy in all surveyed family members. Figure S2: Immunoblots of PHB1, PHB2, MFN1, MFN2, OPA1, DRP1 and GAPDH in PBMCs. Representative immunoblots of PHB1, PHB2, MFN1, MFN2, OPA1, DRP1, and GAPDH in PBMCs from 2 individuals with optic atrophy, who carry PHB1 variant and 2 controls before (0 h) and after H2O2 exposure for the indicated time periods (6 h, 24 h). Figure S3: Immunofluorescence images of PHB2, DRP1 and TOMM20 in PBMCs. The figure displays representative immunofluorescence images of PHB2, DRP1, and TOMM20 in PBMCs from individuals with (P1, P2) and without the PHB1 variant (C1, C2). The fluorescence intensity and the signal distribution of PHB2 show no significant differences between individuals with (P1, P2) and those without the PHB1 variant (C1, C2). The same observation applies to TOMM20 immunofluorescence in the same samples. The fluorescence intensity of DRP1 is lower in both individuals with the PHB1 variant (P1, P2) compared to the control individuals without the PHB1 variant (C1, C2), while the distribution pattern remains comparable across samples. In the same samples, we also observe a lower fluorescence intensity of the TOMM20 signal in individuals with the PHB1 variant (P1, P2) compared to the control individuals without the PHB1 variant (C1, C2). There are no differences in the distribution of the TOMM20 signal between the samples. Figure S4: TEM imaging of mitochondria. TEM imaging was performed in patients who carry the PHB1 variant ( [file CGE-110-165-s001.docx]

Supplementary information

# Table of Contents

[eMethods 3](#_Toc214455099)

[Clinical assessment of patients, recruitment of family members for functional studies and ethical statement 3](#_Toc214455100)

[DNA isolation, exome sequencing, and data analysis 3](#_Toc214455101)

[Family-based linkage analysis 3](#_Toc214455102)

[Isolation of PBMCs and setup of *in vitro* experiments 3](#_Toc214455103)

[Quantification of mtDNA copy number by RT-qPCR 4](#_Toc214455104)

[Immunofluorescence analysis by confocal microscopy 4](#_Toc214455105)

[Antibodies 5](#_Toc214455106)

[Ultrastructural analysis of mitochondria by transmission electron microscopy (TEM) 5](#_Toc214455107)

[Western blot analyses 5](#_Toc214455108)

[RNA-seq analysis 6](#_Toc214455109)

[In silico structural analysis of the PHB Ser147Phe variant 6](#_Toc214455110)

[Quantification and statistical data analysis 6](#_Toc214455111)

[Immunoblotting and immunofluorescence data analysis 7](#_Toc214455112)

[eResults 8](#_Toc214455113)

[Clinical ophthalmic assessment 8](#_Toc214455114)

[Functional characterization of the missense variant in the PHB1 protein 9](#_Toc214455115)

[eDiscussion 11](#_Toc214455116)

[eFigures 14](#_Toc214455117)

[eFigure 1: Genome-wide linkage analysis scan 14](#_Toc214455118)

[eFigure 2: Immunoblots of PHB1, PHB2, MFN1, MFN2, OPA1, DRP1 and GAPDH in PBMCs 15](#_Toc214455119)

[eFigure 3: Immunofluorescence images of PHB2, DRP1 and TOMM20 in PBMCs 16](#_Toc214455120)

[eFigure 4: TEM imaging of mitochondria 18](#_Toc214455121)

[eFigure 5: Relative mitochondrial DNA copy number estimation. 19](#_Toc214455122)

[eFigure 6: OCT showing thinned retinal ganglion cells in the macular area in the patient IV-2 at the age of 5 years. 20](#_Toc214455123)

[eTables 21](#_Toc214455124)

[eTable 1: In silico prediction tools 21](#_Toc214455125)

[eReferences 23](#_Toc214455126)

#

# eMethods

## Clinical assessment of patients, recruitment of family members for functional studies and ethical statement

The study was performed on a Slovenian three-generation family with 4 affected members with optic neuropathy. Initial clinical evaluations confirmed optic neuropathy characterized by progressive vision loss and optic disc pallor were performed at Eye Hospital UMC Ljubljana. All affected subjects were referred to genetic testing. Written informed consent was obtained from all tested subjects.

## DNA isolation, exome sequencing, and data analysis

Blood samples were collected from 4 affected family members and 5 unaffected members, and DNA was extracted according to a standard protocol. Initially, exome sequencing was carried out in 4 affected members and a curated set of genes in the panel Optic neuropathy (nhsgms-panelapp.genomicsengland.co.uk/panels/186/v3.0) was investigated. The variants were classified according to the guidelines of the American College of Medical Genetics and Genomics (ACMG)^1^.

## Family-based linkage analysis

A genome-wide linkage analysis screen was performed by genotyping 3927 markers in 4 affected and 5 unaffected family members. Parametric linkage analysis was performed with the Merlin program, assuming autosomal dominant inheritance with 95% penetrance, disease allele frequency 0.001, and phenocopy rate 0.0, and using the ‘‘affected-only’’ design, which classifies all individuals with no clinical manifestations as ‘‘diagnosis unknown’’.

## Isolation of PBMCs and setup of *in vitro* experiments

Experiments for the functional characterization of the PHB1 variant were performed on peripheral blood mononuclear cells (PBMCs) derived from 2 affected female family members and 2 controls, namely a first-degree male relative and a healthy female subject unrelated to the family. The ages of patients were 47 and 25 years, while the ages of control individuals were 21 (male) and 38 (female) years, respectively.

PBMCs were isolated from single venous blood drawn (collected in Vacutainer® Heparin Tubes) by centrifugation in density gradient medium Lymphoprep ^TM^ (StemCell Technology) following a standard protocol. The isolated PBMCs were then cultured in PB-MAX medium (Gibco) at 37°C and 5% CO_2_ for 7 days, with medium changing every 2-3 days. Before performing qPCR, RNA-seq and Western blot assays, PBMCs were serum-starved (MEM medium (Gibco) only) for 24 h and then for 6h and/or 24h treated with 1000 µM H_2_O_2_ to induce oxidative stress.

## Quantification of mtDNA copy number by RT-qPCR

Real-time quantitative polymerase chain reaction (RT-qPCR) was performed to evaluate mitochondrial DNA (mtDNA) content in PBMC of 2 healthy controls and 2 mutation carriers. Their genomic DNA was isolated from cultivated PBMCs by the QIAamp® DNA Mini Kit (51304, Qiagen) following the manufacturer's manuals. The mtDNA copy number was determined relative to nuclear DNA (nucDNA) copy number according to the protocol of Rooney et al^2^ using SYBR Green chemistry and primers: nuclear gene B2M forward, 5’-TGCTGTCTCCATGTTTGATGTATCT-3’ and reverse, 5'-TCTCTGCTCCCCACCTCTAAGT-3’; mitochondrial gene D-loop forward, TCACCCTATTAACCACTCA-3’ and reverse, 5’-AGACAGATACTGCGACATA-3’; mitochondrial gene ND1 forward, 5’-CACCCAAGAACAGGGTTTGT-3’ and reverse, 5’- TGGCCATGGGTATGTTGTTAA-3’. Each PCR reaction included 6 ng of genomic DNA, 400 nM of each primer, 1x Power SYBR Green PCR Master Mix (Applied Biosystems, Thermo Fisher Scientific) and water in a final volume of 20 µl. Reactions were carried out in 96-well plates using the following cycling parameters: 95 °C for 10 min; 40 cycles of 95°C for 15 s, 60°C for 60 s on the QuantStudio™ 5 Real-Time PCR System (Thermo Fisher Scientific). Each sample was amplified in triplicate and Ct-values obtained from triplicate reactions were averaged. mtDNA contents were calculated using the 2 x 2ΔCt method.

## Immunofluorescence analysis by confocal microscopy

According to a previously described protocol for adhesion and immunostaining of lymphocytes^3^, PBMCs were adhered to cover slips and fixed with ice-cold absolute methanol for 5 min at RT. After fixation, the samples were washed in PBS and blocked in blocking buffer (2,5% BSA (A3059, Sigma Aldrich, Merck) and 2,5% goat serum (G9023, Sigma-Aldrich, Merck KGaA, Darmstadt, Germany) in PBS) for 1h, RT to block non-specific labelling. The samples were then incubated at 37 °C for 2h with the primary antibodies listed in the section Antibodies. After washing in PBS, samples were incubated with appropriate secondary antibodies at room temperature for 1.5 h. After washing in PBS, the samples were mounted in Vectashield mounting medium with 4′,6-diamidino-2-phenylindole (DAPI) (Vector Laboratories, Burlingame, CA, USA) for DNA labelling. The samples were analysed with a confocal laser-scanning microscope LSM 900 (Carl Zeiss MicroImaging GmbH, München, Germany). Images were acquired with a 63× oil immersion objective and the Airyscan 2 detection system. All samples were imaged with the same acquisition settings. Fluorescence intensity was quantified using ZEISS ZEN lite 3.2 software. Fluorescence heterogeneity of PHB1 was quantified in Fiji by measuring the mean fluorescence intensity and standard deviation of PHB1 pixel intensities within individual PBMCs. The coefficient of variation (CV = SD/mean) was calculated for each cell as a measure of spatial signal distribution. For imaging PBMCs were selected randomly for each sample and each image was coded with a number combination that contained no information about the cells or the donor. The images were analysed blindly by another person who had no knowledge of the image coding and scored according to the morphology of the mitochondria in the PBMCs.

## Antibodies

List of used antibodies: anti-prohibitin (Abcam #ab28172), anti-PHB2 (E1Z5A) (Cell Signaling Technology #14085), anti-Mitofusin-1 (D6E2S) (Cell Signaling Technology #14739), anti-Mitofusin-2 (D2D10) (Cell Signaling Technology #9482), anti-OPA1 (BD Transduction Laboratories #612606), anti-DRP1 (Cell Signaling Technology #8570), anti-TOMM20 (Sigma-Aldrich #WH0009804M1, anti-GAPDH (Merck, Sigma-Aldrich #G9545), Goat anti-Rabbit IgG Secondary antibody HRP (Sigma-Aldrich A0545), Goat anti-Mouse IgG Secondary antibody HRP (Abcam #ab6789 Goat anti-Rabbit IgG (H+L) Cross-Adsorbed Secondary Antibody, Alexa Fluor™ 488 (Invitrogen #A-11008) or Goat anti-Mouse IgG (H+L) Cross-Adsorbed Secondary Antibody, Alexa Fluor™ 555 (Invitrogen #A-21422).

## Ultrastructural analysis of mitochondria by transmission electron microscopy (TEM)

Cultured PBMCs were fixed in 2.5% glutaraldehyde in cacodylate buffer (0.1 M, pH 7.0) for 1 hour at RT, centrifuge at 500 x g for 5 min, followed by overnight rinsing in the 0.1 M cacodylate buffer at 4 °C and post-fixed in 2% (w/v) osmium tetroxide for 1 h at room temperature. The samples were then dehydrated in a graded series of ethanol and embedded in Epon (Serva Electrophoresis, Heidelberg, Germany). Ultrathin sections were contrasted with uranyl acetate and lead citrate and examined with a transmission electron microscope (Philips CM100, Tokyo, Japan) equipped with AMT camera (Advanced Microscopy Techniques Corp., Woburn, MA, USA).

## Western blot analyses

Cultured PBMCs were lysed in RIPA buffer (RIPA Lysis Buffer, 10X, Merck 20-188) supplemented with protease (Sigma #P8340) and phosphatase inhibitors (PhosSTOP Roche^TM^, Fisher Scientific). Lysates were clarified by centrifugation at max speed for 10 minutes at 4°C. Equal amounts (usually 20 µg) of proteins obtained from lysed cells were denatured and loaded on sodium dodecyl sulfate polyacrylamide gels. After electrophoresis, the separated proteins from the gel were transferred onto Transfer membraneImmobilon®-P (Merck Millipore, Carl Roth). Protein blots were then incubated with specific primary antibodies listed in the section Antibodies, followed by HRP secondary antibodies and signals were detected by ECL system (Pierce™ ECL Western Blotting Substrate (Thermo Fisher Scientific)). Syngene™ Transilluminator with G:Box Systems (Fisher Scientific) was used for detection and ImageJ software (National Institutes of Health) was used for quantification of signals.

## RNA-seq analysis

Peripheral blood from 2 affected family members and 2 controls was collected directly into Tempus™ blood RNA tubes. A total RNA was isolated using Nucleic acid isolation system QuickGene-810 (FUJIFILM) and QuickGene RNA blood cell kit S following the manufacturer's manuals. Total RNA was quantified by NanoDrop^TM^ 2000/2000c Spectrophotometers (Thermo Fisher Scientific) and the RNA integrity was estimated by Agilent 2100 Bioanalyzer (Agilent Technologies) using RNA 6000 Nano LabChip kit. RNA-Seq libraries and whole transcriptome sequencing (WTS) were performed in the CeGaT GmbH (Tubingen, Germany). A total RNA from cultivated PBMCs was isolated using RNeasy Mini kit (Qiagen) following the manufacturer's manuals. RNA integrity and quantification were evaluated as previously described. RNA-Seq libraries and coding transcriptome sequencing (CTS) were performed in the CeGaT GmbH (Tubingen, Germany).

## In silico structural analysis of the PHB Ser147Phe variant

The structural analysis of the PHB (NM_001281496.1): c.440C>T p.(Ser147Phe) mutation was conducted using a combination of computational modeling and structural inspection tools. The wild-type (WT) and mutated protein structures were predicted using AlphaFold2 (AF2) based on amino acid sequences obtained from the UniProt database (primary accession: P35232). The resulting models were analyzed using PyMOL version 3.1 to visualize structural changes and identify potential interactions.

Protein interactions were examined using the hydrogen bond inspection function in PyMOL to assess hydrogen bonding and steric effects introduced by the Ser147Phe substitution. To further evaluate the pathogenic potential of the given variant, the AlphaMissense algorithm was applied, generating a pathogenicity prediction score of 0.9955 (on a scale from 0 to 1), indicating a high likelihood of functional disruption.

This combined approach allowed for an in-depth assessment of the structural and functional impact of the Ser147Phe pathogenic variant in the PHB protein, including potential loss of hydrogen bonding, steric hindrance, and altered hydrophobic interactions.

## Quantification and statistical data analysis

RNAseq data analysis

RNA sequencing data were aligned to the GRCh37 human reference genome. Gene-level read counts were obtained using the featureCounts function (subread package) with paired-end, strand-specific settings and GTF-based annotation (Ensembl v87). Lowly expressed genes were filtered by retaining genes with CPM ≥1 in at least two samples, and normalization factors were calculated using the trimmed mean of M-values (TMM) method (calcNormFactors, edgeR).

Design matrices were constructed to model the influence of status (Affected vs Unaffected), timepoint (e.g., provocation vs no-provocation), tissue type, and individual variability. Differential expression (DE) analyses were performed using the voom-transformed expression data (limma package), followed by linear modeling (lmFit) and empirical Bayes moderation (eBayes). Comparisons included: affected vs unaffected individuals at baseline and provocation timepoints, provocation vs non-provocation conditions, and blood-specific effects. Significance was defined by adjusted p-values (FDR < 0.05).

Genes with nominal p-values < 0.05 from each DE comparison were subjected to enrichment analysis. Gene symbols were mapped to Entrez IDs using org.Hs.eg.db, and enrichment was performed using KEGG and Gene Ontology (GO) terms via the clusterProfiler package. Significance thresholds were p-value < 0.05 and q-value < 0.2.

We obtained a panel of genes associated with oxidative stress response from the GSEA pathway repository (https://www.gsea-msigdb.org/gsea/msigdb/human/geneset/GOBP_RESPONSE_TO_OXIDATIVE_STRESS). Differentially expressed genes (DEGs) were identified from PBMC transcriptomes comparing the response to stress in patients versus unaffected controls. Genes from the oxidative stress panel overlapping with DEGs from the latter comparison were selected for downstream analysis. Expression data for these genes were extracted from the relevant PBMC samples at baseline and 6-hour timepoints in affected and unaffected individuals. The resulting matrix was z-score normalized by row, and a heatmap was generated using the pheatmap package, with sample annotations and color-coded metadata for visualization.

## Immunoblotting and immunofluorescence data analysis

Data analyses for immunoblotting and immunofluorescence analyses were performed using Microsoft Excel, where two-tailed Student’s t-test was used to compare the differences between two groups (patients versus control cells).

# eResults

## Clinical ophthalmic assessment

A three-generation family is presented (Figure 2) in which 4 members (2 men and 2 women) have been diagnosed with optic neuropathy and followed up regularly for almost 20 years at the Eye Hospital, UMC Ljubljana. Visual acuity (VA), colour vision were tested, visual fields were done, visual electrophysiology testing with pattern electroretinogram (PERG) and pattern-reversal visual evoked potentials (VEP) were performed according to the ISCEV standards. Imaging of the fundus was done, including optical coherence tomography (OCT).

Proband, the eldest male (II-3) was first seen by a local ophthalmologist because of a slowly progressive, profound loss of visual acuity (VA) beginning around the age of 40, first in the left eye (LE) and later in the right eye (RE). When he was examined at the University Eye Hospital, at the age of 61 years, Snellen VA on RE was 0.1cc, and on LE counting fingers (CF) at 1.5 m. Color vision was abnormal (0/15 Ishihara). He had a bilateral central scotoma. Fundus examination revealed optic disc pallor in both eyes. Optical coherence tomography (OCT) showed bilateral atrophy of the retinal nerve fiber layer (RNFL), with atrophy of retinal ganglion cells (RGC) in the macular area. Electrophysiological testing with PERG showed a slight reduction of the P50 component, which probably appeared due to loss of fixation, but there was a marked N95 abnormality with prolonged and reduced pattern-reversal VEP bilaterally, consistent with abnormal functioning of retinal ganglion cells and both optic nerves. At the age of 63 years, his VA deteriorated to CF 1.5m RE and CF 1m LE, Ishihara 0/15 on both eyes.

Proband’s female nephew (III-2) was first seen at the age of 34 years. She had had visual problems since the second year of primary school. Snellen VA was 0.2cc in both RE and LE, and Ishihara was abnormal (2/15) in both eyes. Fundus examination revealed bilateral optic disc pallor. OCT showed bilateral RNFL atrophy. Electrophysiology showed reduced PERG N95 component with delayed peak time and reduced amplitude of the VEP. At follow-up in 2016, peripapillary RNFL thinning was greatest in the superior and inferior quadrants of the disc, with borderline thinning of the temporal and preserved nasal peripapillary RNFL. Progression of the peripapillary RNFL continued, and by 2019, there was complete atrophy of the peripapillary RNFL in both eyes. At her last exam at the age of 44 years, her visual acuity was 0.1cc in both eyes, her colour vision was abnormal (1/15 Ishihara) in both eyes. There was bilateral pallor of the optic nerves. On OCT, there was complete peripapillary RNFL atrophy, as well as atrophy of retinal ganglion cells in the macular area. At her last exam at the age of 44 years her visual acuity was 0.1cc in both eyes, her colour vision was abnormal (1/15 Ishihara) in both eyes. There was bilateral pallor of optic nerves. On OCT there was complete peripapillary RNFL atrophy, as well as atrophy of retinal ganglion cells in the macular area.

The male nephew of the proband (III-3) was first seen at the Eye Hospital at the age of 33 years due to bilateral poor visual acuity since youth. Snellen VA was 0,4cc from both RE and LE. Color vision was abnormal (1/15 Ishihara). Optic discs were pale temporally. MRI of the head was normal. Electrophysiological testing showed abnormal wave-shape of the PERG N95 component, with delayed peak time and still normal VEP amplitudes, consistent with optic neuropathy. In 2016, VA decreased slightly to RE 0.32, LE 0.2. He also has a sensorineural hearing impairment.

Female (IV-1) was first seen at the age of 8 years (2006). Snellen VA was 0.8cc in the RE and LE. There were temporally pale optic discs seen at her fundus. Electrophysiology showed a normal PERG N95 component, indicating preserved functioning of the retinal ganglion cells. VEP showed normal amplitude, but markedly delayed peak time, consistent with abnormal conduction along the optic nerves. At the age of 16 years, Snellen VA was 0.6 in RE and 0.4 in LE. Color vision was slightly abnormal (Ishihara 11/15 RE and 9/15 LE). There was some reduced sensitivity centrally in the visual field. Fundus examination revealed temporal pallor of the optic discs, OCT showed bilateral peripapillary RNFL atrophy, and inner retinal atrophy in the macular region with a tendency to progression. Head CT was normal. Electrophysiology also showed progression, with decreased PERG N95 component delayed VEP peak time and slight relative reduction of the amplitudes. At the age of 23 years (2021), VA had decreased to 0.4 cc in RE and 0.3 cc in LE, and there was a bilateral central scotoma in the visual field. Fundus examination revealed bilateral optic disc pallor. OCT showed RNFL atrophy of the optic nerves, there was atrophy of the RGC in the macular area. Clinical characteristics of affected family members are represented in Table 1. Other family members had no problems with their vision (II-2, III-5, IV-3 and IV-4); the youngest male member, born in 2019 (IV-2), was examined in 2025 at the age of 5 years. His visual acuity was still normal, however his optic nerves were slightly paler as would be expected for his a age and there were signs of an early optic neuropathy seen on his OCT (thinned retinal ganglion cells seen in the macular area, see eFigure 6).

## Functional characterization of the missense variant in the PHB1 protein

To assess how the heterozygous missense variant c.440C>T (p.Ser147Phe) in the *PHB1* gene impacts the protein’s structure, function, and interactions, which may lead to disease or altered biological processes, we first made homology modeling. Results of comparative modeling suggest the hydrogen bond involving Ser147 is abolished, creating areas of structural strain or instability. Distances between interacting atoms exceed the optimal range for hydrogen bonds (3.8–4.9 Å compared to the ideal 2.5–3.5 Å), indicating weaker or no bonding. Due to the bulky phenylalanine ring, all the neighbouring amino acids change the orientation as depicted by the alignment of wild type to the mutant structure. The change in orientation might severely influence the bonding pattern between side chains (Figure 3B).

Constraint metrics from gnomAD (v4.1.1) for PHB1 demonstrate a pronounced depletion of missense variation (observed/expected = 0.56, 95% CI 0.50–0.63; Z = 3.54), whereas synonymous variants occur at the expected rate (o/e = 0.99, 95% CI 0.86–1.14; Z = 0.07), indicating selective constraint specifically against amino-acid–altering variants. Predicted loss-of-function variants are also reduced (o/e = 0.35, 95% CI 0.21–0.59; pLI = 0.81), although the relative enrichment of missense constraint compared with neutral variation suggests that disease is more likely mediated by deleterious missense changes rather than simple haploinsufficiency. Consistent with this interpretation, the DOMINO tool (https://domino.iob.ch/), which predicts the mode of inheritance using gene-level features, estimates a probability of autosomal dominant inheritance of 0.586 for *PHB1* (LDA score = 0.585; classification: either dominant or recessive but leaning toward AD). Together, these data support a dominant disease mechanism driven by heterozygous missense variants, consistent with the heterozygous missense variant identified here as the possible cause of optic atrophy.

Second, we explored if the identified variant in *PHB1* might impact mtDNA copy number. Comparison of the mtDNA copy number of two samples carrying the *PHB1* variant with 2 controls, determined by qPCR in PBMCs, showed no significant difference in mtDNA content between the samples studied (eFigure 5).

Third, to gain insight into gene expression changes potentially associated with the missense variant in the *PHB1* gene, we performed RNA sequencing (RNA-seq) on cultured PBMCs from two variant carriers and two control individuals without the variant, both before and after the induction of oxidative stress. Additionally, RNA-seq was conducted on whole blood samples from the same individuals. Due to the small sample size, no genes met the criteria for differential expression using a stringent statistical threshold (p < 0.01), and therefore multiple comparison correction was not applied. Under physiological conditions (in both blood and PBMC samples), differentially expressed genes included non-coding RNAs, pseudogenes, chemokine receptor genes, genes encoding interferon-stimulated proteins, ribosomal protein genes, and others with a range of functions. Notably, many of these genes were associated with immune responses, cellular signaling, gene regulation, and stress response pathways. Following oxidative stress induction, more pronounced expression changes were observed in PBMCs of patients harboring the identified *PHB1* variant.

# eDiscussion

Autosomal dominant optic atrophy (ADOA) (OMIM #165500) is an inherited optic neuropathy that typically starts in early childhood with progressive loss of retinal ganglion cells (RGCs), the neurons responsible for transmitting visual signals from the retina to the brain^6^. Most cases are linked to pathogenic variants in the *OPA1* gene, which encodes a mitochondrial fusion protein and has been implicated in over 70% of ADOA cases^7,8^. Pathogenic variants in *OPA1* impair mitochondrial function and disorganize mitochondrial cristae structures. Clinical evidence shows that energy-demanding cells, such as RGCs in the papillo-macular bundle, which have a high density of mitochondria in the unmyelinated portion of their axons, are particularly vulnerable to mitochondrial dysfunction. Additionally, the constant exposure of the central retina to oxidative stress from light passing through the eye further exacerbates this vulnerability. Taken together, mitochondrial defects caused by pathogenic *OPA1* variants probably associate lower energy efficiency with higher ROS production, along with damage induced by light exposure, may contribute to the increased degeneration of RGCs in the central retina^6^.

Besides OPA1, the structural integrity of mitochondria is maintained by a variety of proteins, one of the most important being Prohibitin 1 (PHB1). PHB1 is a nuclear-encoded protein that resides in the inner mitochondrial membrane, where it forms a high-molecular-weight complex with its close family member, PHB2. Together, PHB1 and PHB2 act as a scaffold that is essential for a range of critical mitochondrial functions, including involvement in stability/proteolysis of OXPHOS system subunits and mitochondrial protein translation ^9^. PHB1 is essential for life as germline deletion of the PHB gene leads to embryonic lethality ^10^. Probably less detrimental effect presents missense mutations, and one of it is a heterozygous missense variant in the *PHB1* gene, which was detected in a three-generation family with four individuals affected by optic neuropathy. Whole-exome and whole genome-based linkage mapping identified a heterozygous missense variant c.440C>T (p.Ser147Phe) in the *PHB1* gene as a possible genetic cause of optic atrophy in this family. Since a missense mutation can have a range of effects, from altering macromolecular stability to disrupting macromolecular interactions and cellular localization^11,12^ and the majority of in silico prediction tools predict a pathogenic effect of p.Ser147Phe variant in the PHB1 protein (eTable 1), we first performed an in silico structural modelling. The results showed that the p.Ser147Phe substitution in the PHB protein disrupts protein stability and function, possibly due to loss of hydrogen bonding. To validate these findings, we performed *in vitro* experiments using peripheral blood mononuclear cells (PBMCs) from two affected individuals and two healthy controls. WB analysis revealed altered band patterns for PHB1 in the patient samples compared to controls, including under oxidative stress treatment (H_2_O_2_). The appearance of split bands of PHB1 protein could indicate a post-translational modification (PTM) of the protein. Changes in PTMs may affect the protein’s subcellular localization^13^, a hypothesis we tried to explore through immunofluorescence staining of the PHB1 protein. Immunofluorescence staining and confocal microscopy revealed no significant difference in the fluorescence intensity of PHB1 between patient and control cells. However, the distribution of PHB1 was notably different: in patient cells, PHB1 showed a more heterogeneous cytoplasmic distribution, whereas in controls the signal appeared more spatially confined, with a tendency towards perinuclear enrichment. This was further supported by the higher coefficient of variation measured across individual cells. This shift in localization warrants further investigation.

Next, we assessed whether this PHB1 localization change affects mitochondrial dynamics, specifically mitochondrial fusion and fission processes, which are crucial for mitochondrial function and morphology. The balance between fusion and fission controls mitochondrial number, shape and size^14^ and the active role of PHB1 in OPA1-mediated membrane fusion was recently revealed^15^. While there was no significant difference in mitochondrial DNA content between the patient and control samples, WB analysis of patient PBMCs showed reduced levels of MFN1 and MFN2, key proteins involved in mitochondrial fusion, and a decreased L-OPA1/S-OPA1 ratio compared to controls. Taken together, these changes suggest that the PHB1 variant may impair mitochondrial fusion, but likely has a milder effect on cell proliferation and apoptosis compared to PHB2 deletion. The latter leads to the selective loss of long isoforms of OPA1, resulting in aberrant cristae morphogenesis, impaired cellular proliferation, and reduced resistance to apoptosis^16^.

To investigate mitochondrial fission, we examined the levels of dynamin-related protein 1 (DRP1), the primary mediator of mitochondrial fission. While WB analysis showed no differences in DRP1 expression between patient and control PBMCs, immunofluorescence microscopy revealed an increased intensity of the DRP1 signal in the perinuclear area in patient cells, suggesting that DRP1 might be recruited from the cytosol to the mitochondria where it regulates mitochondrial morphology, dynamics and consequently effect mitochondrial function^14^ in response to the mutation. The differences in mitochondrial morphology were tried to discover by transmission electron microscopy, but no major differences were observed between patient and control PBMCs. This shows that p.Ser147Phe in PHB1 protein has probably less detrimental effect on mitochondria ultrastructure in comparison to the ultrastructure of *PHB2* deleted cells mitochondria^16^ or Opa1^−/−^ cells mitochondria, where mitochondria predominantly display sparse cristae with decreased nucleoid-cristae proximity, that are partly rescued by OPA1 stable expression^17^.

Since manipulation of *PHB1* expression in PC12 cells has identified PHB1 as a positive modulator of mitochondrial function under oxidative stress^18^, we investigated the biological pathways that are differentially activated or repressed under stress conditions in cells expressing the *PHB1* variant. Patients' and control PBMCs cells were exposed to H_2_O_2_, and RNA-seq analysis revealed a set of genes involved in the cellular stress response that are repressed in the patient cells compared to control cells. These results suggest that the *PHB1* gene variant might contribute to a diminished cellular response under stress conditions. This impaired stress response could make particularly vulnerable cells, such as RGCs, more susceptible to damage, leading to the observed optic atrophy.

However, future studies should include detailed assessments of mitochondrial shape, number, and length to determine whether these features differ between groups. Additionally, a more thorough investigation of DRP1 phosphorylation sites, which regulate mitochondrial fission, will be important for understanding the mutation’s impact on mitochondrial dynamics. Future studies should also explore the role of mitochondrial fission and mitophagy in the selective removal of damaged mitochondria or the progression of apoptosis^19^, although these analyses were beyond the scope of the current study due to limited tissue samples.

This study provides the first evidence suggesting that *PHB1* could be a novel gene linked to optic atrophy. However, the exact mechanism by which the identified variant affects the protein and its function remains unclear. While the current evidence is not sufficient to definitively establish causality, our findings provide a valuable foundation for future research into the role of *PHB1* in hereditary optic neuropathies and other related human diseases.

# eFigures


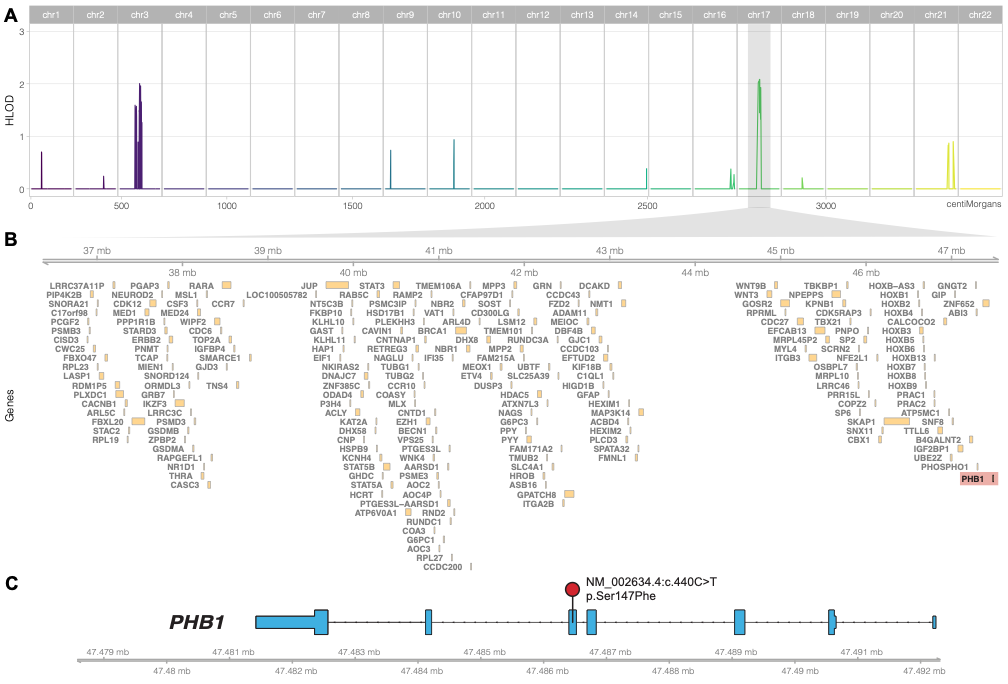


## eFigure 1: Genome-wide linkage analysis scan

(A) Results of the genome-sequencing-based linkage scan in the family. An interval on chromosome 17 (chr17: 36.5-47.5 Mb) with a maximum LOD score of 2.1 was identified by analysis of co-segregating SNP markers. (B) The region on chromosome 17 with the highest linkage score was surveyed for candidate genes and variants, including the PHB1 gene. (C) A heterozygous missense variant c.440C>T (p.Ser147Phe) in exon 4 was observed to perfectly cosegregate with the optic atrophy in all surveyed family members.


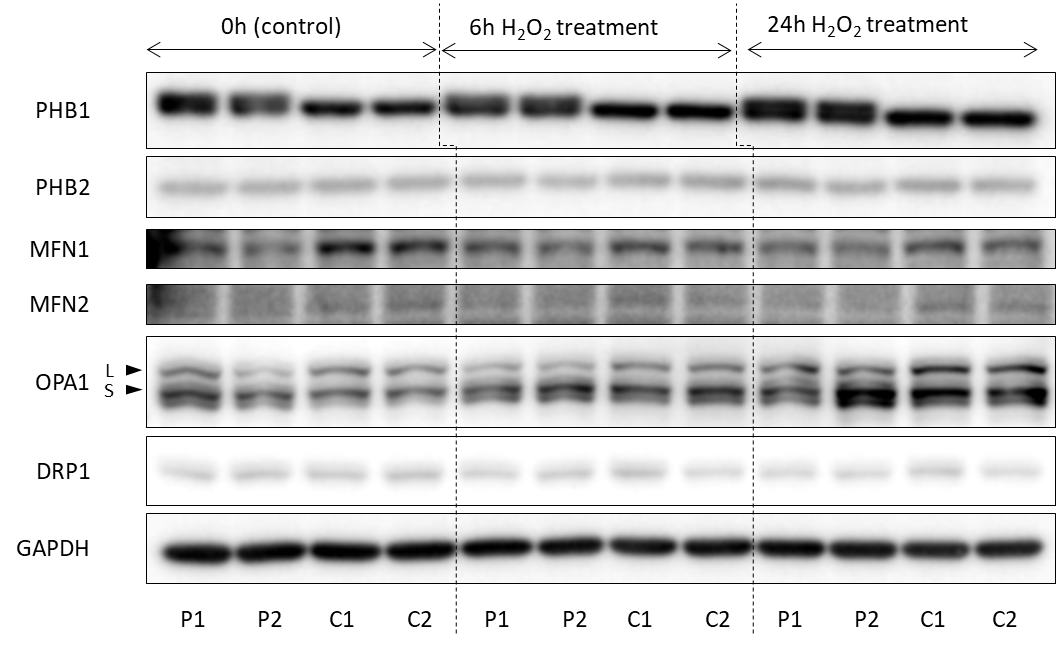


## eFigure 2: Immunoblots of PHB1, PHB2, MFN1, MFN2, OPA1, DRP1 and GAPDH in PBMCs

Representative immunoblots of PHB1, PHB2, MFN1, MFN2, OPA1, DRP1 and GAPDH in PBMCs from 2 individuals with optic atrophy, who carry PHB1 variant and 2 controls before (0h) and after H_2_O_2_ exposure for the indicated time periods (6h, 24h).


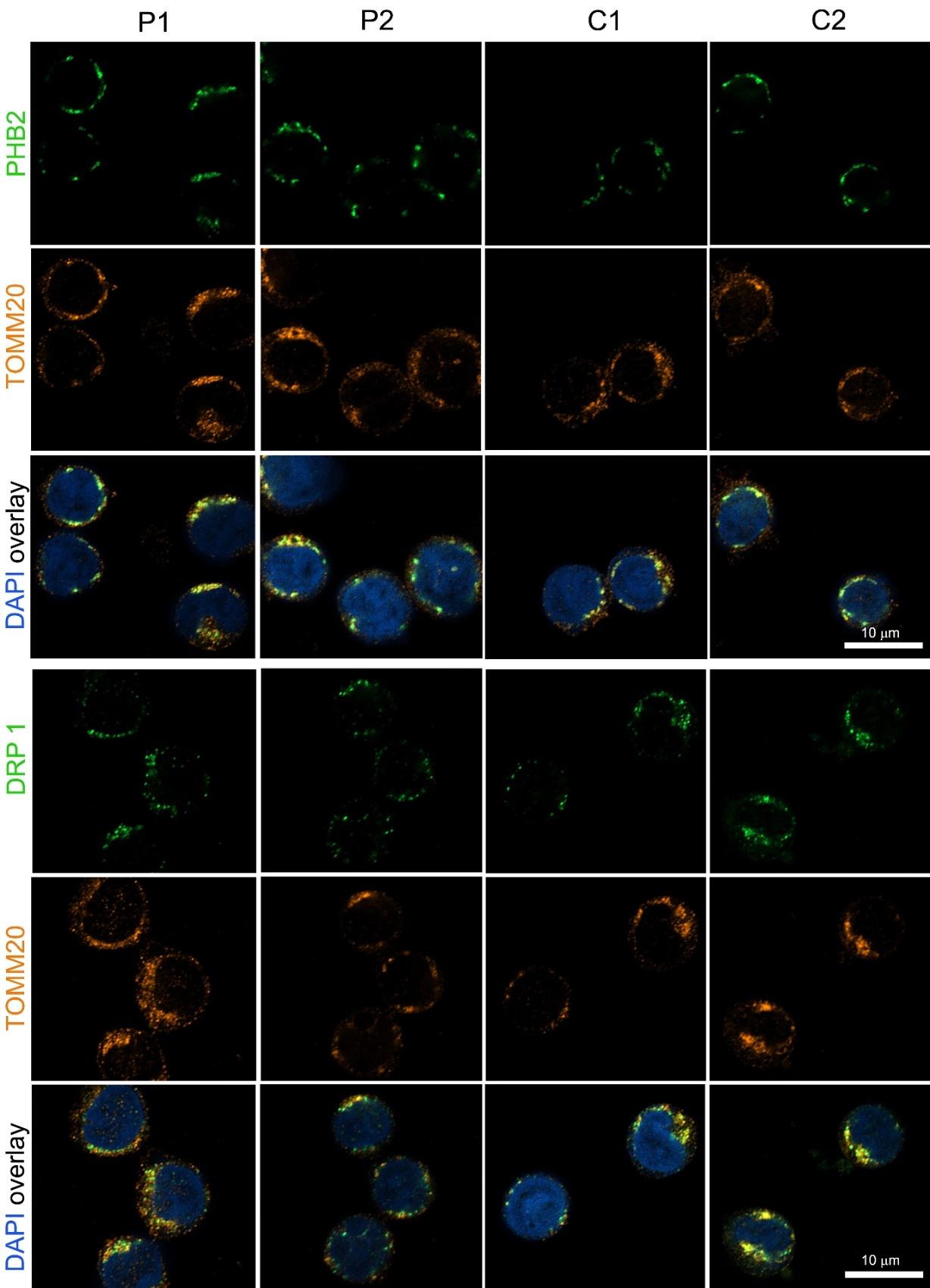


## eFigure 3: Immunofluorescence images of PHB2, DRP1 and TOMM20 in PBMCs

The figure displays representative immunofluorescence images of PHB2, DRP1, and TOMM20 in PBMCs from individuals with (P1, P2) and without the PHB1 variant (C1, C2). The fluorescence intensity and the signal distribution of PHB2 show no significant differences between individuals with (P1, P2) and those without the PHB1 variant (C1, C2). The same observation applies to TOMM20 immunofluorescence in the same samples. The fluorescence intensity of DRP1 is lower in both individuals with the PHB1 variant (P1, P2) compared to the control individuals without the PHB1 variant (C1, C2), while the distribution pattern remains comparable across samples. In the same samples, we also observe a lower fluorescence intensity of the TOMM20 signal in individuals with the PHB1 variant (P1, P2) compared to the control individuals without the PHB1 variant (C1, C2). There are no differences in the distribution of the TOMM20 signal between the samples.


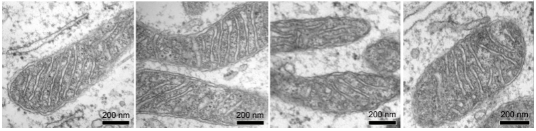


P1 P2 C1 C2

## eFigure 4: TEM imaging of mitochondria

TEM imaging was performed in patients who carry the PHB1 variant (P1 and P2) and controls without the variant (C1 and C2). The ultrastructure of the mitochondria shows no significant differences between the patients and controls.


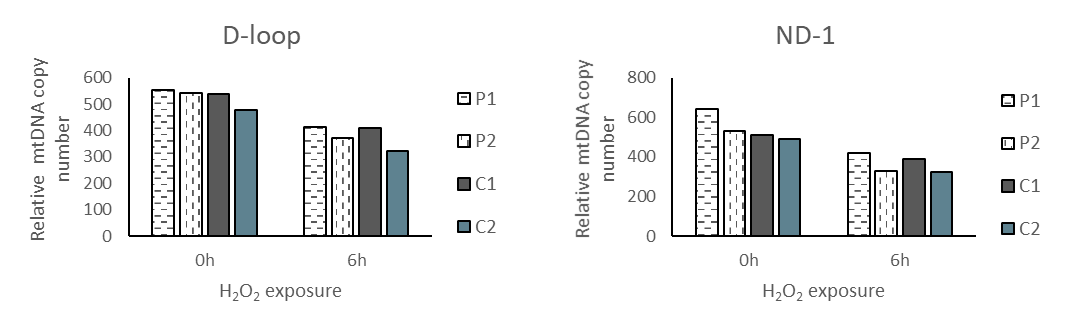


## eFigure 5: Relative mitochondrial DNA copy number estimation.

Relative mitochondrial DNA copy number was estimated using RT-qPCR measurement of mitochondrial D-loop and ND1 (relative to nuclear B2M gene) in PBMCs from 2 subjects carrying the PHB1 variant (P1, P2) and two control subjects without the variant (C1, C2), both before and after inducing oxidative stress with 1000 µM H_2_O_2_ for 6 hours.

## eFigure 6: OCT showing thinned retinal ganglion cells in the macular area in the patient IV-2 at the age of 5 years.


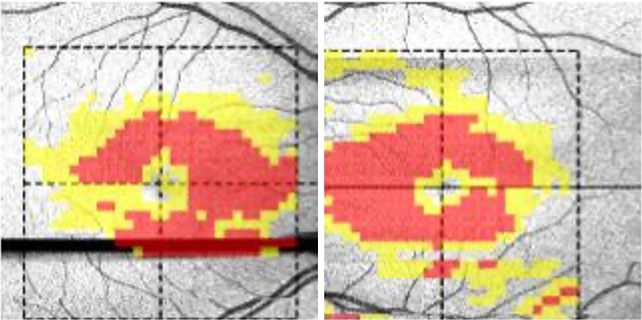


##

# eTables

## eTable 1: In silico prediction tools

| In silico prediction tool | Prediction |
| --- | --- |
| Meta scores | 13 (Very Strong ≥ 8 points) |
| BayesDel addAF | Pathogenic Strong (0.5233) |
| BayesDel noAF | Pathogenic Moderate (0.5139) |
| MetaRNN | Pathogenic Moderate (0.9268, 0.9268, 0.9268, 0.9268, 0.9268, 0.9268, 0.9268) |
| MetaSVM | Pathogenic Moderate ( 0.9396) |
| REVEL | Pathogenic Moderate (0.945, 0.945, 0.945, 0.945, 0.945) |
| MetaLR | Pathogenic Supporting (0.8868) |
| AlphaMissense | Pathogenic Strong (0.9955) |
| DEOGEN2 | Pathogenic Strong (0.9844, 0.9844, 0.9844, 0.8512, 0.8574) |
| FATHMM-MKL | Pathogenic Moderate (0.9938) |
| MutPred | Pathogenic Moderate (0.727, 0.727, 0.727, 0.727, 0.727, 0.727, 0.727) |
| MVP | Pathogenic Moderate (0.9831, 0.9831, 0.9831, 0.9831, 0.9831, 0.9831, 0.9831) |
| FATHMM-XF | Pathogenic Supporting (0.9313) |
| LIST-S2 | Pathogenic Supporting (0.9827, 0.9827, 0.9827, 0.9827, 0.9827) |
| LRT | Pathogenic Supporting (0) |
| M-CAP | Pathogenic Supporting (0.4126) |
| Mutation assessor | Pathogenic Supporting (3.075, 3.075, 3.075) |
| PrimateAI | Pathogenic Supporting (0.8798) |
| PROVEAN | Pathogenic Supporting (-5.53, -5.73, -5.73, -5.73, -5.74) |
| SIFT | Pathogenic Supporting (0, 0, 0, 0, 0) |
| BLOSUM | Uncertain (-5) |
| DANN | Uncertain (0.9969) |
| EIGEN | Uncertain (0.5261) |
| EIGEN PC | Uncertain (0.5889) |
| FATHMM | Uncertain (-3.54, -3.54, -3.54, -3.54, -3.54) |
| MutationTaster | Uncertain (1, 1) |
| SIFT4G | Uncertain (0.011, 0.011, 0.011, 0.012, 0.013) |

# eReferences

[1] Richards S, Aziz N, Bale S, Bick D, Das S, Gastier-Foster J, et al. Standards and guidelines for the interpretation of sequence variants: A joint consensus recommendation of the American College of Medical Genetics and Genomics and the Association for Molecular Pathology. *Genetics in Medicine*. 2015;17(5):405-424. doi:10.1038/gim.2015.30

[2] Rooney JP, Ryde IT, Sanders LH, Howlett E V., Colton MD, Germ KE, et al. PCR based determination of mitochondrial DNA copy number in multiple species. *Methods in Molecular Biology*. 2015;1241:23-38. doi:10.1007/978-1-4939-1875-1_3,

[3] Tsang M, Gantchev J, Ghazawi FM, Litvinov I V. Protocol for adhesion and immunostaining of lymphocytes and other non-adherent cells in culture. *Biotechniques*. 2017;63(5):230-233. doi:10.2144/000114610

[4] Sun J, Ren H, Wang J, Xiao X, Zhu L, Wang Y, et al. CHAC1: a master regulator of oxidative stress and ferroptosis in human diseases and cancers. *Front Cell Dev Biol*. 2024;12:1458716. doi:10.3389/fcell.2024.1458716

[5] Rao G, Murphy B, Dey A, Dwivedi SKD, Zhang Y, Roy RV, et al. Cystathionine beta synthase regulates mitochondrial dynamics and function in endothelial cells. *FASEB Journal*. 2020;34(7):9372-9392. doi:10.1096/fj.202000173R

[6] Lenaers G, Neutzner A, Le Dantec Y, Jüschke C, Xiao T, Decembrini S, et al. Dominant optic atrophy: Culprit mitochondria in the optic nerve. *Prog Retin Eye Res*. 2021;83. doi:10.1016/j.preteyeres.2020.100935

[7] Ferré M, Bonneau D, Milea D, Chevrollier A, Verny C, Dollfus H, et al. Molecular screening of 980 cases of suspected hereditary optic neuropathy with a report on 77 novel OPA1 mutations. *Hum Mutat*. 2009;30(7). doi:10.1002/humu.21025

[8] Almind GJ, Ek J, Rosenberg T, Eiberg H, Larsen M, LuCamp LC, et al. Dominant optic atrophy in Denmark - report of 15 novel mutations in OPA1, using a strategy with a detection rate of 90%. *BMC Med Genet*. 2012;13. doi:10.1186/1471-2350-13-65

[9] Signorile A, Sgaramella G, Bellomo F, De Rasmo D. Prohibitins: A Critical Role in Mitochondrial Functions and Implication in Diseases. *Cells*. 2019;8(1). doi:10.3390/cells8010071

[10] Sanz MA, Tsang WY, Willems EM, Grivell LA, Lemire BD, Van der Spek H, et al. The mitochondrial prohibitin complex is essential for embryonic viability and germline function in Caenorhabditis elegans. *Journal of Biological Chemistry*. 2003;278(34):32091-32099. doi:10.1074/jbc.M304877200

[11] Flores E, Acharya N, Castañeda CA, Sukenik S. Single-point mutations in disordered proteins: Linking sequence, ensemble, and function. *Curr Opin Struct Biol*. 2025;91:102987. doi:10.1016/J.SBI.2025.102987<SPAN

[12] Stefl S, Nishi H, Petukh M, Panchenko AR, Alexov E. Molecular mechanisms of disease-causing missense mutations. *J Mol Biol*. 2013;425(21):3919-3936. doi:10.1016/j.jmb.2013.07.014

[13] Schofield LC, Dialpuri JS, Murshudov GN, Agirre J. Post-translational modifications in the Protein Data Bank. *Acta Crystallogr D Struct Biol*. 2024;80(9):647-660. doi:10.1107/s2059798324007794

[14] Chan DC. Mitochondrial Dynamics and Its Involvement in Disease. *Annual Review of Pathology: Mechanisms of Disease*. 2020;15:235-259. doi:10.1146/annurev-pathmechdis-012419-032711

[15] Ban T, Kuroda K, Nishigori M, Yamashita K, Ohta K, Koshiba T. Prohibitin 1 tethers lipid membranes and regulates OPA1-mediated membrane fusion. *Journal of Biological Chemistry*. 2025;301(1). doi:10.1016/j.jbc.2024.108076

[16] Merkwirth C, Dargazanli S, Tatsuta T, Geimer S, Löwer B, Wunderlich FT, et al. Prohibitins control cell proliferation and apoptosis by regulating OPA1-dependent cristae morphogenesis in mitochondria. *Genes Dev*. 2008;22(4):476-488. doi:10.1101/gad.460708

[17] Macuada J, Molina-Riquelme I, Vidal G, Pérez-Bravo N, Vásquez-Trincado C, Aedo G, et al. OPA1 and disease-causing mutants perturb mitochondrial nucleoid distribution. *Cell Death Dis*. 2024;15(11):870. doi:10.1038/s41419-024-07165-9

[18] Anderson CJ, Kahl A, Qian L, Stepanova A, Starkov A, Manfredi G, et al. Prohibitin is a positive modulator of mitochondrial function in PC12 cells under oxidative stress. *J Neurochem*. 2018;146(3):235-250. doi:10.1111/jnc.14472

[19] Wang S, Long H, Hou L, Feng B, Ma Z, Wu Y, et al. The mitophagy pathway and its implications in human diseases. *Signal Transduct Target Ther*. 2023;8(1). doi:10.1038/S41392-023-01503-7,
